# Validation of diagnosis codes to identify side of colon in an electronic health record registry

**DOI:** 10.1186/s12874-019-0824-7

**Published:** 2019-08-19

**Authors:** Patricia Luhn, Deborah Kuk, Gillis Carrigan, Nathan Nussbaum, Rachael Sorg, Rebecca Rohrer, Melisa G. Tucker, Brandon Arnieri, Michael D. Taylor, Neal J. Meropol

**Affiliations:** 1 Genentech, Inc, South San Francisco, CA USA; 2 Flatiron Health, New York, NY USA; 3 Real World Data-Science (RWD-S) Genentech, a Member of the Roche Group 1 DNA Way, South San Francisco, CA 94080 USA

**Keywords:** Diagnosis code, Metastatic colorectal cancer, Electronic medical record

## Abstract

**Background:**

The use of real-world data to generate evidence requires careful assessment and validation of critical variables before drawing clinical conclusions. Prospective clinical trial data suggest that anatomic origin of colon cancer impacts prognosis and treatment effectiveness. As an initial step in validating this observation in routine clinical settings, we explored the feasibility and accuracy of obtaining information on tumor sidedness from electronic health records (EHR) billing codes.

**Methods:**

Nine thousand four hundred three patients with metastatic colorectal cancer (mCRC) were selected from the Flatiron Health database, which is derived from de-identified EHR data. This study included a random sample of 200 mCRC patients. Tumor site data derived from International Classification of Diseases (ICD) codes were compared with data abstracted from unstructured documents in the EHR (e.g. surgical and pathology notes). Concordance was determined via observed agreement and Cohen’s kappa coefficient (κ). Accuracy of ICD codes for each tumor site (left, right, transverse) was determined by calculating the sensitivity, specificity, positive predictive value (PPV), and negative predictive value (NPV), and corresponding 95% confidence intervals, using abstracted data as the gold standard.

**Results:**

Study patients had similar characteristics and side of colon distribution compared with the full mCRC dataset. The observed agreement between the ICD codes and abstracted data for tumor site for all sampled patients was 0.58 (κ = 0.41). When restricting to the 62% of patients with a side-specific ICD code, the observed agreement was 0.84 (κ = 0.79). The specificity (92–98%) of structured data for tumor location was high, with lower sensitivity (49–63%), PPV (64–92%) and NPV (72–97%). Demographic and clinical characteristics were similar between patients with specific and non-specific side of colon ICD codes.

**Conclusions:**

ICD codes are a highly reliable indicator of tumor location when the specific location code is entered in the EHR. However, non-specific side of colon ICD codes are present for a sizable minority of patients, and structured data alone may not be adequate to support testing of some research hypotheses. Careful assessment of key variables is required before determining the need for clinical abstraction to supplement structured data in generating real-world evidence from EHRs.

**Electronic supplementary material:**

The online version of this article (10.1186/s12874-019-0824-7) contains supplementary material, which is available to authorized users.

## Background

Historically, prospective randomized clinical trials have served as the “gold standard” for evidence generation in oncology. Given that only a small percentage of cancer patients take part in clinical research studies [1], there is increasing interest in leveraging the data contained in administrative and clinical databases for patients treated outside of clinical trials, as these data can provide guidance for treatment decisions. Such real-world data have the potential to be more representative of patients in routine practice, given that clinical trials tend to enroll highly selected patients who are younger and have fewer comorbidities. Furthermore, real-world data can supplement the results of prospective clinical trials in settings where accrual is difficult due to uncommon clinical or genomic selection criteria. Recently, the Twenty-first Century Cures Act [2] and United States Food and Drug Administration 2018 Goals [3] both highlighted the imperative to understand how real-world data can be optimally used to improve health.

The data contained in electronic health records (EHRs) afford an important opportunity to test hypotheses regarding patterns of care and outcomes in a broadly representative sample of cancer patients. EHR data are characterized by date and may not require third-party primary data collection. However, the impact of real-world data is dependent upon the reliability of specific data elements, their completeness, and the ability to ensure and trace their provenance [4]. Thus, the promise of EHR data can only be realized if each data point is carefully assessed and validated before clinical conclusions are drawn.

As an example of the research application of EHR data, we sought to validate in a real-world setting recent clinical findings from a group of prospective clinical trials in patients with metastatic colorectal cancer (mCRC). Historically, the clinical development of systemic therapies for mCRC has not distinguished patients based on the location of the tumor within the bowel. However, recent analyses have suggested that anatomical side of the colon from which a tumor arises is a prognostic and predictive indicator of survival [5–9]. These studies have indicated that CRCs arising from the left or right side of the colon differ significantly in their clinical characteristics and gene expression profiles [10–13], with right-sided tumors being associated with a worse prognosis [14–16]. Therapeutic outcome also may differ by tumor side, with several analyses reporting differences in benefit with epidermal growth factor receptor and vascular endothelial growth factor antibodies in left- vs right-sided mCRC tumors [5, 6]. These findings led to a recent international expert panel recommendation that primary tumor location be included as an essential data element in the design and reporting of colon cancer clinical trials [17]. As an initial step in seeking to replicate these findings in a real-world population, we undertook a formal analysis of the ability to obtain information about tumor sidedness from billing codes (International Classification of Disease [ICD] 9/10) in EHRs. The overall goal of this study was to determine the feasibility of using structured diagnostic codes to determine tumor location for patients with mCRC. The formal validation approach described herein may be broadly applied to other clinical contexts where data points from EHRs are being considered for use in outcomes research.

## Methods

### Data source

This validation study was conducted using the nationwide Flatiron Health database, a longitudinal, demographically and geographically diverse database derived from de-identified EHR data. The Flatiron Health database includes data from over 265 cancer clinics, comprised of both community and academic oncology clinics, representing more than 2 million US cancer patients available for analysis. The de-identified patient-level data in the EHRs includes structured data (e.g. billing codes, laboratory measurements, visits, and prescribed drugs) and unstructured data curated via technology-enabled chart abstraction from physicians’ notes and other unstructured documents (e.g. physician progress notes, pathology reports).

### Patient selection

From the broader Flatiron Health EHR-derived database, a cohort of mCRC patients was created. Patients were selected for an ICD-code of colon or rectal cancer (153.x, 154.x, C18x, C19x, C20x, or C21x), at least two clinic visits in the Flatiron network that occurred on or after January 1, 2013, and clinical documentation of mCRC. Patients lacking relevant unstructured documents in the Flatiron Health database for abstraction were excluded. Of 9403 patients with confirmed metastatic colon cancer, a random sample cohort of 200 patients who met the above criteria was included in this study. The random sample was selected using a random number generator with a specified seed so that the list of patients is reproducible. As the current analysis focused on side of colon, patients with a confirmed diagnosis of metastatic rectal cancer were excluded from the validation study.

### Identification of tumor location

ICD codes were compared with location identified through human abstraction of unstructured data to establish the quality of ICD-defined tumor location. For both ICD-defined and abstracted tumor location variables, tumors were classified as left side (splenic flexure, descending colon, sigmoid colon, rectosigmoid junction), right side (cecum, ascending colon, hepatic flexure), or transverse (transverse colon).

### Identification of tumor location based upon structured data

Data captured in the Flatiron Health EHR-derived database include ICD, 9th and 10th revisions (ICD9 and ICD10; see Table 5 in Appendix) for diagnoses [18]. Whereas some codes can differentiate CRC tumor origin (i.e. ICD9 153.1/ICD10 C18.4: Malignant neoplasm of transverse colon, ICD9 153.7/ICD10 C18.5: Malignant neoplasm of splenic flexure), there is also an unspecified code (ICD9 153.9/ICD10 C18.9: Malignant neoplasm of the colon, unspecified site) that can be used by physicians.

ICD9/10 codes were available from the diagnosis table in the EHR database and were used to classify patients. The full list of codes and categories used is listed in Table 5 in Appendix: A. The date of the ICD code closest to the initial diagnosis date was used to assign side of colon with the following considerations: if a patient had multiple ICD codes that indicated different sides on the same date, and if this date was closest to the diagnosis date, the patient was categorized as having CRC in multiple sites of the colon. If one of the codes was an unspecified code, it was dropped and the specific code was used to classify the patient (e.g. “Left colon, Unspecified colon” became “Left colon”). For patients with no abstracted initial diagnosis date, the first relevant ICD code was selected.

### Identification of tumor location based on chart abstraction

In order to establish the quality of ICD-defined tumor location, ICD codes were compared with location identified through human abstraction of unstructured data. Centrally trained abstractors reviewed all relevant unstructured documents included in the patients’ EHR, including pathology reports, physician notes, and surgical notes to identify evidence of the side of colon. To classify a patient, abstractors looked for terms such as “left colon” or “right colon,” as well as the specific sites within the colon, as described in Table 5 in Appendix: A.

### Statistical methods

Patient characteristics were summarized using counts and percentages for categorical variables, and medians and interquartile ranges for continuous variables, for the full mCRC dataset (9403 patients) and the 200 randomly selected participants in our validation study. Concordance between structured ICD codes and abstracted diagnosis was determined via observed percent agreement and Cohen’s kappa coefficient (κ). The concordance analysis assumed no gold standard. Accuracy of ICD codes was determined by calculating the sensitivity, specificity, positive and negative predictive values, and corresponding 95% confidence intervals, using the abstracted data as the gold standard. “Unspecified colon side” in the unstructured data was treated as “No” for all of these analyses.

## Results

Baseline characteristics for patients in this study (N = 200) were similar to patients in the full mCRC dataset for all variables examined (Table 1). Half of the validation study patients were male (50%), and more than half were aged 65 and older (59%), and had stage IV mCRC at initial diagnosis (54%). An additional 28% had stage III CRC at initial diagnosis. Site-specific ICD codes were available for 5940 (63%) patients in the parent cohort (Table 2).

**Table 1:** Patient characteristics of full EDM registry patients and 200 randomly selected study patients

Baseline characteristics, n (%)	Sampled patients *N* = 200	Parent cohort *N* = 9403
Age at metastatic diagnosis (years), median (IQR)	67.0 (57.0–76.0)	66.0 (56.0–75.0)
Age at metastatic diagnosis category, years		
18–34	2 (1.0)	118 (1.3)
35–49	25 (12.5)	1003 (10.7)
50–64	55 (27.5)	3162 (33.6)
≥ 65	118 (59.0)	5120 (54.5)
Sex		
Female	100 (50.0)	4385 (46.6)
Male	100 (50.0)	5017 (53.4)
Other/Unknown	0	1 (0.01)
Region		
Northeast	53 (26.5)	2630 (28.0)
Midwest	40 (20.0)	1627 (17.3)
South	71 (35.5)	3340 (35.5)
West	30 (15.0)	1470 (15.6)
Other/unknown	6 (3.0)	336 (3.6)
Practice type		
Community	188 (94.0)	8923 (94.9)
Academic	12 (6.0)	480 (5.1)
Stage at diagnosis		
0–I	5 (2.5)	199 (2.1)
II	18 (9.0)	993 (10.6)
III	55 (27.5)	2247 (23.9)
IV	107 (53.5)	5627 (59.8)
Not documented	15 (7.5)	337 (3.6)
Tumor site: Colon	200 (100.0)	9403 (100.0)
Side of colon (from abstracted data)		
Left side	99 (49.5)	NA
Right side	67 (33.5)	NA
Transverse colon	12 (6.0)	NA
Unspecified	22 (11.0)	NA

*EDM* Electronic data mart, *NA* not applicable

**Table 2:** Comparison of patient and clinical characteristics based on presence of specific ICD codes

Characteristic, n (%)	Colon cancer patients with specific ICD codes N = 5940	Colon cancer patients without specific ICD codes N = 3463
Age at metastatic diagnosis (years), median (IQR)	66.0 (56.0–75.0)	66.0 (57.0–75.0)
Age at metastatic diagnosis, years		
18–34	72 (1.2)	46 (1.3)
35–49	650 (10.9)	353 (10.2)
50–64	2014 (33.9)	1148 (33.2)
≥ 65	3204 (53.9)	1916 (55.3)
Sex		
Female	2771 (46.6)	1614 (46.6)
Male	3169 (53.4)	1848 (53.4)
Unknown	0 (0)	1 (< 0.1)
Region		
Northeast	1329 (22.4)	826 (23.9)
Midwest	1214 (20.4)	412 (11.9)
South	2263 (38.1)	1074 (31.0)
West	826 (13.9)	643 (18.6)
Other/unknown	308 (5.2)	508 (14.7)
Year of mCRC diagnosis		
2011	457 (7.7)	350 (10.1)
2012	795 (13.4)	602 (17.4)
2013	1218 (20.5)	838 (24.2)
2014	1359 (22.9)	792 (22.9)
2015	1547 (26.0)	679 (19.6)
2016	564 (9.5)	202 (5.8)
Practice type		
Community	5851 (98.5)	3072 (88.7)
Academic	89 (1.5)	391 (11.3)
Stage at diagnosis		
0–I	115 (1.9)	84 (2.4)
II	629 (10.6)	364 (10.5)
III	1408 (23.7)	839 (24.2)
IV	3639 (61.3)	1988 (57.4)
Unknown	149 (2.5)	188 (5.4)
Tumor site		
Left side^a^	3061 (51.5)	NA
Right side	2377 (40.0)	NA
Transverse colon	477 (8.0)	NA
Multiple sides	25 (0.4)	NA
Number of visits post diagnosis, median (Q1, Q3)		
Any	31 (11, 64)	31 (10, 70)
Lab	1 (0, 6)	1 (0, 5)
Treatment	14 (2, 34)	12 (1, 35)
Office	8 (3, 18)	9 (3, 20)
Other	0 (0, 0)	0 (0, 0)
Non-facility	0 (0, 0)	0 (0, 0)
Radiology	1 (1, 1)	1 (1, 1)
Missing	21 (0.4)	21 (0.6)
Line of therapy (ever in database), including maintenance		
1 L	4864 (81.9)	2729 (78.8)
2 L	2252 (37.9)	1341 (38.7)
3 L	986 (16.6)	642 (18.5)
4 L	399 (6.7)	265 (7.7)
1 L treatment regimens (non-maintenance)		
FOLFOX	2537 (52.2)^b^	1367 (50.1)^b^
FOLFIRI	903 (18.6)^b^	533 (19.5)^b^
FOLFOXIRI	45 (0.9)^b^	31 (1.1)^b^
Bevacizumab-containing	2736 (56.2)^b^	1485 (64.4)^b^
Biomarker status		
KRAS tested	4004 (67.4)	2216 (64.0)
KRAS Positive	925 (41.1)^c^	449 (43.1)^c^
NRAS tested	1070 (18.0)	466 (13.5)
NRAS Positive	36 (4.6)^c^	13 (4.2)^c^
BRAF tested	1333 (22.4)	648 (18.7)
BRAF Positive	101 (11.7)^c^	38 (10.3)^c^

*1 L* First-line, *FOLFIRI* Leucovorin/5-fluorouracil/irinotecan, *FOLFOX* Leucovorin/5-fluorouracil/oxaliplatin, *FOLFOXIRI* Leucovorin/5-fluorouracil/oxaliplatin/irinotecan, *ICD* International Classification of Diseases, *mCRC* Metastatic colorectal cancer, *NA* Not applicable, *Q1* Quarter 1

^a^Includes patients with ICD codes for rectal cancer. ^b^Percentage is based on number of patients who have a first line of therapy. ^c^Percentage is based on number of patients who have a record of being tested

When patients with unspecified ICD codes were excluded from the analysis, the distribution of side of colon using ICD9/10 codes was similar to the distribution observed using the abstracted tumor site. Of the 200 study patients, 50% had a left-sided tumor, 34% had a right-sided tumor, and 6% had a transverse tumor, based on abstracted data (Table 1 and Table 3). Approximately 4% (n = 8) of patients were considered to have rectal cancer based on ICD codes; however, through chart abstraction these patients had a confirmed diagnosis of colon cancer. Thus, this discrepancy represents misclassification of these patients based on ICD codes alone.

**Table 3:** Sampled patients with side identified by ICD code or by abstraction

Tumor location, n (%)	Side identified by ICD code		Side identified by abstraction (n = 200)
	Including unspecified ICD codes (n = 200)	Excluding unspecified ICD codes (n = 124)	
Left colon only	70 (35)	70 (56)	99 (49.5)
Right colon only	35 (17.5)	35 (28)	67 (33.5)
Transverse colon only	10 (5)	10 (8)	12 (6)
Unspecified colon site only	76 (38)	–	22 (11)
Rectum	8 (4)	8 (6)	0
Right colon and transverse colon	1 (0.5)	1 (0.8)	0

*ICD* International Classification of Diseases

When all 200 study patients were considered, concordance was moderate between the structured (ICD) data and the unstructured (abstracted) data, with an observed agreement of 0.58 (κ = 0.41). When patients who were classified as unspecified or rectal in the structured data were removed, the observed agreement was 0.84 (κ = 0.79). Seventy-six (38%) patients were classified as “unspecified” using ICD codes, and 63 of these (83%) had the side identified through abstraction. As shown in Table 4, specificity of structured data for tumor location was high, ranging from 92 to 98%. Sensitivity, negative predictive value, and positive predictive value were of lower performance, ranging from 49–63%, 72–97%, and 64–92%, respectively. When patients with non-specific side of colon ICD codes were removed, sensitivity improved to ~ 80% for all tumor locations. Similar estimates were observed when stratified by stage at initial diagnosis (Stage I-III vs. Stage IV) (Additional file 1: Tables S1–S4).

**Table 4:** Accuracy of ICD codes^a^ in sampled patients

Accuracy of ICD codes, % (95% CI)	Left	Right	Transverse	Right/Transverse
Sensitivity	63 (52, 72)	49 (37, 62)	58 (29, 84)	52 (40, 63)
Specificity	92 (85, 96)	98 (93, 99)	98 (94, 99)	96 (90, 98)
Positive predictive value	89 (78, 95)	92 (76, 98)	64 (32, 88)	89 (76, 96)
Negative predictive value	72 (63, 79)	79 (72, 85)	97 (94, 99)	75 (68, 82)

*CI* Confidence interval, *ICD* International Classification of Diseases

^a^See Table 5 in Appendix A

In an effort to identify potential biases regarding the likelihood that ICD coding for tumor location was present, we compared the clinical characteristics of those patients who had specific diagnosis codes and those who did not. There were no differences in age, stage, sex, or treatment distributions between these two cohorts (Table 2). A gradual increase in the use of specific ICD codes was observed over time, with 57% of patients diagnosed in 2011 having a specific ICD code, increasing to 74% of patients diagnosed in 2016, and a higher proportion of use of non-specific ICD codes was seen in academic centers compared with community centers; however, the number of academic sites was small compared to community centers.

## Discussion

This study demonstrates that billing codes are a highly reliable indicator of tumor location, when the specific location code is entered in the EHR. For a sizable minority of mCRC patients, non-specific colon cancer ICD codes are captured in the EHR; thus, structured data for these patients do not indicate tumor side of colon. In these cases, chart abstraction can increase the completeness. If studies are restricted to patients with specific ICD codes, there would likely be minimal bias introduced as the patients with and without specific ICD codes were similar with respect to demographic and clinical characteristics.

A few limitations for this study exist. Although chart abstraction was considered the gold standard, it is subject to errors introduced by abstractors potentially mis-reporting information or by inaccurate information being recorded in the unstructured parts of the EHR. However, chart abstraction is the accepted gold standard for validation studies from administrative claims and other databases, such as EHRs. Additionally, billing codes are collected for the purposes of reimbursement, not for research. Thus, a bias may exist if there are reimbursement incentives based on charges for the treatment based on tumor site. Furthermore, there may be variation in how billing codes are assigned and recorded at the centers in the Flatiron network; however, we did not observe any systematic differences based on centers, with the exception of a higher proportion of patients without specific codes being treated at academic centers. Further studies are needed to validate whether these results are representative of a wider range of data sources, including sources from outside of the US where billing coding practices may differ.

Our analysis demonstrates that ICD codes adequately characterize side of colon for use in studying outcomes for left- versus right-sided colon tumors following specific therapies. However, certain other research questions, e.g. characterizing very small populations such as BRAF-mutant mCRC patients by variables including primary tumor site, may require a side of colon variable with greater completeness of specific side of colon data. The high specificity of structured data suggests that this augmentation of ICD codes with chart abstracted data may, in some situations, be targeted to only those patients with non-specific CRC ICD codes. For other situations, such as creation of a matched cohort with tumor side as a covariate, abstracting tumor side for all patients in a cohort may be warranted to optimize the quality of the variable.

## Conclusions

Overall, these analyses demonstrate the rigor necessary to characterize an EHR-based variable in terms of reliability and completeness, before engaging in formal testing of clinical hypotheses that could be practice-changing. Such methodological assessments are necessary before conducting large-scale research using variables generated from EHRs.

### Additional file

Additional file 1: **Table S1.** Distribution of side identified by ICD code or abstraction for patients with Stage IV disease at diagnosis. **Table S2.** Distribution of side identified by ICD code or abstraction for patients with Stage I-III disease at diagnosis. **Table S3.** Accuracy of ICD codes for patients with Stage IV disease at diagnosis. **Table S4.** Accuracy of ICD codes for patients with Stage I-III disease at diagnosis (DOCX 19 kb)

## Data Availability

The data that support the findings of this study are available from Flatiron Health; however, restrictions apply to the availability of these data, which are subject to the de-identification requirements of the Health Insurance Portability and Accountability Act of 1996 (HIPAA) and implementing regulations, as amended. Practically speaking, in order to share select data and data elements, it will be necessary to first define the methods of storage, transmission, access rights, and scope of intended use prior to making any such data available, and an agreement memorializing the same and applicable re-identification restrictions will be required for the purposes of ensuring compliance with the data license, de-identification, data protection specifications and requirements under HIPAA. Please refer any questions or requests regarding data used in this manuscript to Melisa Tucker (mtucker@flatiron.com) and include Dr. Neal Meropol (nmeropol@flatiron.com) on the email request.

## Appendix

**Table 5:** ICD-9/10 colorectal mappings

Diagnosis	ICD-9 code	ICD-10 code
Right colon (ascending colon)		
Hepatic flexure	153.0	C18.3
Cecum	153.4	C18.0
Ascending colon	153.6	C18.2
Transverse colon		
Transverse colon	153.1	C18.4
Left colon (descending colon)		
Descending colon	153.2	C18.6
Sigmoid colon	153.3	C18.7
Splenic flexure	153.7	C18.5
Rectosigmoid junction	154.0	C19
Unspecified colon site		
Colon unspecified	153.9	C18.9
Malignant neoplasm of appendix vermiformis	153.5	N/A
Malignant neoplasm of appendix	N/A	C18.1
Malignant neoplasm of other specified sites of large intestine	153.8	N/A
Malignant neoplasm of overlapping sites of colon	N/A	C18.8
Rectum		
Rectum	154.1	C20
Malignant neoplasm of other sites of rectum, rectosigmoid junction, and anus	154.8	N/A
Malignant neoplasm of anus, unspecified	N/A	C21.0
Malignant neoplasm of anal canal	N/A	C21.1
Malignant neoplasm of overlapping sites of rectum, anus and anal canal	N/A	C21.8

*ICD* International Classification of Diseases, *N/A* Not applicable

## Abbreviations

CRC: Colorectal cancer
EHR: Electronic health record;
ICD: International Classification of Diseases
mCRC: Metastatic colorectal cancer
NPV: Negative predictive value
PPV: Positive predictive value

## References

[1] Murthy VH, Krumholz HM, Gross CP (2004). Participation in cancer clinical trials; race-, sex-, and age-based disparities. JAMA..

[2] United States Food and Drug Administration (2017). Real-world data and evidence in drug development.

[3] United States Food and Drug Administration (2018). PDUFA reauthorization performance goals and procedures fiscal years 2018 through 2022.

[4] Miksad RA, Abernethy AP (2018). Harnessing the power of real-world evidence (RWE): a checklist to ensure regulatory-grade data quality. Clin Pharmcol Ther.

[5] Arnold D, Lueza B, Douillard JY (2017). Prognostic and predictive value of primary tumour side in patients with RAS wild-type metastatic colorectal cancer treated with chemotherapy and EGFR directed antibodies in six randomised trials. Ann Oncol.

[6] Loupakis F, Yang D, Yau L, et al. Primary tumor location as a prognostic factor in metastatic colorectal cancer. J Natl Cancer Inst. 2015;107:1–9. 10.1093/jnci/dju427 PMC4565528 25713148

[7] Venook AP (2016). Metastatic colorectal cancer: lessons learned, future possibilities. J Natl Compr Canc Netw.

[8] Venook AP (2017). Right-sided vs left-sided colorectal cancer. Clin Adv Hematol Oncol.

[9] Weiss Jennifer M., Pfau Patrick R., O'Connor Erin S., King Jonathan, LoConte Noelle, Kennedy Gregory, Smith Maureen A. (2011). Mortality by Stage for Right- Versus Left-Sided Colon Cancer: Analysis of Surveillance, Epidemiology, and End Results–Medicare Data. Journal of Clinical Oncology.

[10] Meza R, Jeon J, Renehan AG (2010). Colorectal cancer incidence trends in the United States and United Kingdom: evidence of right- to left-sided biological gradients with implications for screening. Cancer Res.

[11] Missiaglia E, Jacobs B, D’Ario G (2014). Distal and proximal colon cancers differ in terms of molecular, pathological, and clinical features. Ann Oncol.

[12] Benedix F, Kube R, Meyer F (2010). Comparison of 17,641 patients with right- and left-sided colon cancer: differences in epidemiology, perioperative course, histology, and survival. Dis Colon Rectum.

[13] Meguid RA, Slidell MB, Wolfgang CL (2008). Is there a difference in survival between right- versus left-sided colon cancers?. Ann Surg Oncol.

[14] Petrelli Fausto, Tomasello Gianluca, Borgonovo Karen, Ghidini Michele, Turati Luca, Dallera Pierpaolo, Passalacqua Rodolfo, Sgroi Giovanni, Barni Sandro (2017). Prognostic Survival Associated With Left-Sided vs Right-Sided Colon Cancer. JAMA Oncology.

[15] Modest DP, Schulz C, von Weikersthal LF (2014). Outcome of patients with metastatic colorectal cancer depends on the primary tumor site (midgut vs. hindgut): analysis of the FIRE1-trial (FuFIRI or mIROX as first-line treatment). Anticancer Drugs.

[16] Yahagi M, Okabayashi K, Hasegawa H (2016). The worse prognosis of right-sided compared with left-sided colon cancers: a systematic review and meta-analysis. J Gastrointest Surg.

[17] Goey KKH, Sørbye H, Glimelius B (2018). Consensus statement on essential patient characteristics in systemic treatment trials for metastatic colorectal cancer: supported by the ARCAD group. Eur J Cancer.

[18] Centers for Disease Control and Prevention (CDC) International Classification of Diseases. https://www.cdc.gov/nchs/data/dvs/icd10fct.pdf. Accessed 17 July 2018.

