# Supplementary material for: Validation of diagnosis codes to identify side of colon in an electronic health record registry
Source: BMC Med Res Methodol. 2019 Aug 19;19:177. doi: 10.1186/s12874-019-0824-7 (PMC6700780; doi:10.1186/s12874-019-0824-7)
Supplement: Supplementary file 1 — Table S1. Distribution of side identified by ICD code or abstraction for patients with Stage IV disease at diagnosis. Table S2. Distribution of side identified by ICD code or abstraction for patients with Stage I-III disease at diagnosis. Table S3. Accuracy of ICD codes for patients with Stage IV disease at diagnosis. Table S4. Accuracy of ICD codes for patients with Stage I-III disease at diagnosis (DOCX 19 kb) [file 12874_2019_824_MOESM1_ESM.docx]

**Table S1** Distribution of side identified by ICD code or abstraction for patients with Stage IV disease at diagnosis

| Tumor location, n (%) | Side identified by ICD code | | Side identified by abstraction  (n=107) |
| --- | --- | --- | --- |
|  | Including unspecified ICD codes (n=107) | Excluding unspecified ICD codes (n=67) |  |
| Left colon only | 36 (33.6) | 36 (53.7) | 51 (47.7) |
| Right colon only | 20 (18.7) | 20 (29.9) | 36 (33.6) |
| Transverse colon only | 7 (6.5) | 7 (10.4) | 8 (7.5) |
| Unspecified colon site only | 40 (37.4) | - | 12 (11.2) |
| Rectum | 4 (3.7) | 4 (6) | 0 |

**Table S2** Distribution of side identified by ICD code or abstraction for patients with Stage I-III disease at diagnosis

| Tumor location, n (%) | Side identified by ICD code | | Side identified by abstraction  (n=93) |
| --- | --- | --- | --- |
|  | Including unspecified ICD codes (n=93) | Excluding unspecified ICD codes (n=57) |  |
| Left colon only | 34 (36.6) | 34 (59.6) | 48 (51.6) |
| Right colon only | 15 (16.1) | 15 (26.3) | 31 (33.3) |
| Transverse colon only | 3 (3.2) | 3 (5.3) | 4 (4.3) |
| Unspecified colon site only | 36 (38.7) | - | 10 (10.8) |
| Rectum | 4 (4.3) | 4 (7.0) | 0 |

**Table S3** Accuracy of ICD codes for patients with Stage IV disease at diagnosis

| Accuracy of ICD codes, % (95% CI) | Left | Right | Transverse | Right/Transverse |
| --- | --- | --- | --- | --- |
| Sensitivity | 61 (46, 74) | 53 (36, 69) | 62 (26, 90) | 57 (41, 71) |
| Specificity | 91 (80, 97) | 99 (91, 100) | 98 (92, 100) | 97 (88, 99) |
| Positive predictive value | 86 (70, 95) | 95 (73, 100) | 71 (30, 95) | 93 (74, 99) |
| Negative predictive value | 72 (60, 82) | 80 (70, 88) | 97 (91, 99) | 76 (65, 85) |

**Table S4** Accuracy of ICD codes for patients with Stage I-III disease at diagnosis

| Accuracy of ICD codes, % (95% CI) | Left | Right | Transverse | Right/Transverse |
| --- | --- | --- | --- | --- |
| Sensitivity | 65 (49, 77) | 45 (28, 64) | 50 (15, 85) | 46 (29, 63) |
| Specificity | 93 (81, 98) | 97 (88, 99) | 98 (91, 100) | 95 (85, 99) |
| Positive predictive value | 91 (75, 98) | 88 (60, 98) | 50 (15, 85) | 84 (60, 96) |
| Negative predictive value | 71 (58, 82) | 78 (67, 86) | 98 (91, 100) | 74 (63, 83) |
